# Tyrosine phosphorylation and protein degradation control the transcriptional activity of WRKY involved in benzylisoquinoline alkaloid biosynthesis

**DOI:** 10.1038/srep31988

**Published:** 2016-08-24

**Authors:** Yasuyuki Yamada, Fumihiko Sato

**Affiliations:** 1 Division of Integrated Life Science, Graduate School of Biostudies, Kyoto University, Kyoto, 606-8502, Japan

## Abstract

Benzylisoquinoline alkaloids (BIQ) are among the most structurally diverse and pharmaceutically valuable secondary metabolites. A plant-specific WRKY-type transcription factor, CjWRKY1, was isolated from *Coptis japonica* and identified as a transcriptional activator of BIQ biosynthesis. However, the expression of *CjWRKY1* gene alone was not sufficient for the activation of genes encoding biosynthetic enzymes. Here, we report the importance of post-translational regulation of CjWRKY1 in BIQ biosynthesis. First, we detected the differential accumulation of CjWRKY1 protein in two cell lines with similar *CjWRKY1* gene expression but different levels of accumulated alkaloids. Further investigation of the WRKY protein identified the phosphorylation of the WRKYGQK core domain at Y115. The CjWRKY^Y115E^ phosphorylation-mimic mutant showed loss of nuclear localization, DNA-binding activity, and transactivation activity compared to wild-type CjWRKY1. Rapid degradation of the CjWRKY1 protein was also confirmed following treatment with inhibitors of the 26S proteasome and protease inhibitors. The existence of two independent degradation pathways as well as protein phosphorylation suggests the fine-tuning of CjWRKY1 activities is involved in the regulation of biosynthesis of BIQs.

Higher plants produce a large variety of low molecular weight secondary metabolites, including nitrogen-containing alkaloids, which are often used as pharmaceuticals^1^. Alkaloid biosynthesis, including the relevant biosynthetic enzymes and genes, has been intensively investigated^2^,^3^,^4^, but the regulation of alkaloid biosynthesis, especially by transcription factors, is largely unknown because only a limited number of plant species and cells produce certain types of alkaloids, making analysis difficult. Recently, however, WRKY and basic helix-loop-helix transcription factors in benzylisoquinoline alkaloids (BIQ) biosynthesis and some WRKY, bHLH and AP2/ERF proteins in the biosynthesis of nicotine and monoterpenoid indole alkaloids biosynthesis have been isolated^5^,^6^,^7^,^8^,^9^,^10^.

Plants-specific WRKY transcription factors are one of the most important regulators in defence responses, development, and senescence^11^,^12^,^13^. They all contain the 60-amino acid WRKY domain, which has a highly conserved amino acid sequence, WRKYGQK, at the N-terminal end, and a zinc finger motif at the C-terminal end. The WRKY domain specifically recognizes the W-box DNA motif (TTGACC/T)^13^. The WRKY family can be divided into three groups based on the structures; (i) Group I has two WRKY domains and a C2H2-type zinc-finger motif, (ii) Group II has a single WRKY domain and the C2H2 zinc-finger, and can be further divided into five subgroups, (iii) Group III has a single WRKY domain and a zinc finger structure of C2HC^13^. The first WRKY identified in alkaloid biosynthesis, CjWRKY1, was isolated from *C. japonica* cells in the biosynthesis of BIQ-type alkaloids^5^ (see BIQ biosynthetic pathway, Supplementary Fig. S1), and belongs to Group IIc.

Transient RNAi of *CjWRKY1* clearly decreased the expression of BIQ biosynthetic enzyme genes, including *tyrosine decarboxylase* (*TYDC*), (*S*)*-norcoclaurine synthase* (*NCS*), (*S*)*-norcoclaurine 6-O-methyltransferase* (*6OMT*), (*S*)*-coclaurine-N-methyltransferase* (*CNMT*), (*S*)*-N-methylcoclaurine 3*′*-hydroxylase* (*CYP80B2*), (*S*)*-3*′*-hydroxy-N-methylcoclaurine-4*′*-O-methyltransferase* (*4*′*OMT*), *berberine bridge enzyme* (*BBE*), and *canadine synthase* (*CYP719A1*), in high BIQ-producing Cj156-S cells. Over-expression of *CjWRKY1* enhanced the transcript levels of the above-mentioned genes in Cj156-S cells. These results confirmed that CjWRKY1 is a specific and general transcriptional activator of BIQ biosynthesis in Cj156-S cells^5^. However, we also found that high expression of *CjWRKY1* transcripts was not correlated with the expression of BIQ biosynthetic enzyme genes in low BIQ-producing CjY cells^5^. Thus, we attempted to clarify why high *CjWRKY1* gene expression did not induce the expression of target biosynthetic genes in CjY cells.

Post-translational modification seems to be one of the key regulatory mechanisms of transcription factors involved in the regulation of plant processes. In fact, some WRKY proteins are reported to be post-translationally regulated by protein phosphorylation, protein-protein interaction and protein turnover^14^,^15^. Here, we report that the post-translational regulation of CjWRKY1, i.e., tyrosine phosphorylation and the 26S proteasome-mediated and non-mediated protein degradation, would play a crucial role in the regulation of CjWRKY1 activity in BIQ biosynthesis.

## Results

### Tyrosine phosphorylation of CjWRKY1

**Figure 1:**
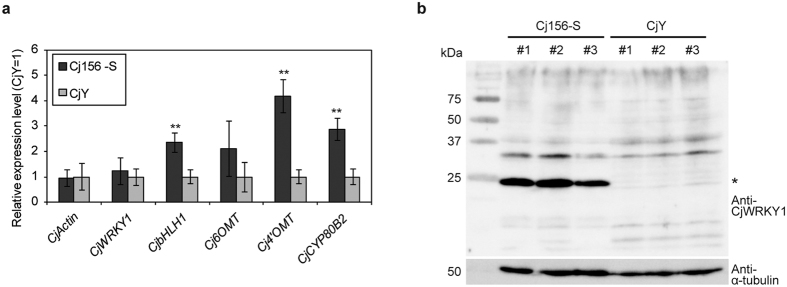
Expression of *CjWRKY1* and the biosynthetic enzyme genes in Cj156-S and CjY cells. (**a**) Transcripts of *CjWRKY1* and the other biosynthetic enzyme genes in 2-week-cultured cells were measured by quantitative RT-PCR. The relative expression levels were estimated by the standard curve method with three biological replicates and standardized with the *α-tubulin* gene as an internal control. The average value of the CjY was set as 1. The data are shown as the mean ± s.d., **p < 0.01, Student’s *t*-test. (**b**) CjWRKY1 accumulation in three independent cultures grown for 2 weeks. An asterisk indicates CjWRKY1 protein. Left axis indicates the size of reference proteins. kDa; molecular mass in kilodaltons.

We first prepared specific antibodies against a CjWRKY1 peptide and characterized the expression of CjWRKY1 in both Cj156-S and CjY cells (Fig. 1). Immunoblotting analysis clearly detected the accumulation of CjWRKY1 in Cj156-S cells but not in CjY cells. Thus, we next considered the possibility of post-translational modification of CjWRKY1 in *C. japonica* cells.

We examined the modification of the CjWRKY1 protein using Cj156-S cells because CjWRKY1 in CjY cells was too unstable for analysis. Recombinant CjWRKY1-sGFP proteins expressed in Cj156-S protoplasts were recovered via co-immunoprecipitation (Co-IP) with anti-GFP antibody-bound microbeads and analysed by liquid chromatography coupled with tandem-mass spectrometry (LC-MS/MS) after trypsin digestion. MassMatrix analysis of peptide fragments of CjWRKY1 showed phosphorylation of CjWRKY1 at Tyr115, which is part of the WRKYGQK core sequence (Supplementary Table S1, Supplementary Fig. S2).

**Figure 2:**
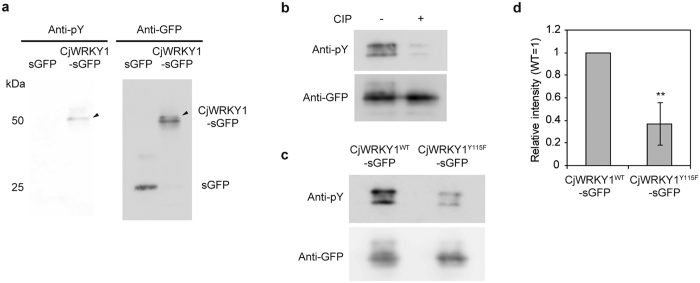
Tyrosine phosphorylation of CjWRKY1. (**a**) CjWRKY1-sGFP or sGFP (as a control) protein was expressed in *C. japonica* 156-S protoplasts, immunoprecipitated with anti-GFP microbeads and immunodetected with anti-pY and anti-GFP antibodies. Arrows indicate the bands of tyrosine-phosphorylated CjWRKY1-sGFP. Left axis indicates the size of reference proteins. kDa; molecular mass in kilodaltons, pY; phospho-tyrosine. (**b**) CIP treatment of co-IP samples. Co-IP samples were treated without (−) or with (+) CIP before immunoblot analysis. (**c**) Immunodetection of CjWRKY1^WT^-sGFP or CjWRKY1^Y115F^-sGFP with anti-pY antibodies. (**d**) The signal intensity of Fig. 2c was quantified by ImageJ software. The value is the average of results from three independent experiments. The data are represented as the mean ± s.d., *p < 0.05, Student’s *t*-test. The experiments repeated at least three times in a and b showed similar results.

The tyrosine phosphorylation of CjWRKY1 was further confirmed with anti-phospho-tyrosine (pY) antibodies (Fig. 2a). The phosphorylation signal was detected as a slightly slower moving band during immunoblot analysis with anti-GFP antibodies after the removal of anti-pY antibodies (Fig. 2a). The relatively faint phosphorylation signal, less than 40% compared to total signal intensity (Supplementary Fig. S3), suggest that phosphorylation of CjWRKY1 was not strong in Cj156-S cells or that there was rapid turnover of phosphorylated proteins as discussed below. Calf intestine alkaline phosphatase (CIP) treatment of IP proteins confirmed that the signal was derived from the phosphorylated CjWRKY1-sGFP (Fig. 2b). These data demonstrate the tyrosine phosphorylation of the CjWRKY1 protein in *C. japonica* cells.

Phosphorylation of CjWRKY1 at Y115 was also characterized using mutant CjWRKY1^Y115F^ with a Phe substitution for Y115. A much weaker signal was observed with immunoprecipitated CjWRKY1^Y115F^-sGFP with anti-pY antibodies compared to wild-type CjWRKY1^WT^-sGFP (Fig. 2c,d), confirming the phosphorylation of Y115. However, the remaining signal also suggested the possibility of phosphorylation at other Tyr residue(s) in CjWRKY1.

### Functional characterization of phosphorylation of CjWRKY1

**Figure 3:**
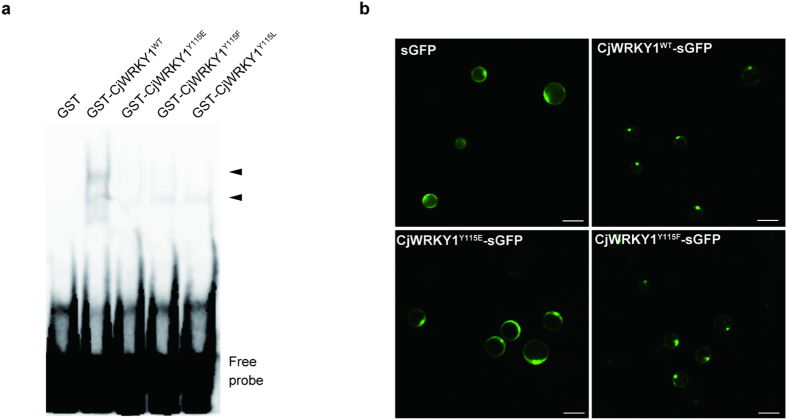
The phosphorylation of CjWRKY1 at Tyr 115 affected the DNA binding and nuclear localization of the CjWRKY1 protein. (**a**) EMSA of GST-CjWRKY1 mutated at Tyr115. Arrows indicate the shifted bands corresponding to the protein-DNA complexes. (**b**) Subcellular localization of WT and mutant CjWRKY1-sGFP proteins in Cj156-S protoplasts. Scale bars represent 50 μm.

To investigate the functional role of phosphorylation of CjWRKY1 at Y115, mutant CjWRKY1^Y115E^ with a Glu residue at Y115 to generate a phosphorylation mimic as well as another mutant, CjWRKY^Y115L^, with a Leu substitution to generate a dephosphorylated peptide mimic were prepared. Then, their DNA-binding activities to WRKY target cis-elements (W-box) were characterized in comparison to wild-type CjWRKY1. Electrophoretic mobility shift assays (EMSA) using an oligonucleotide probe containing three tandem copies of the W-box found in the *CYP80B2* gene promoter (Supplementary Fig. S4) showed decreased DNA binding activities of all mutants, while the CjWRKY1^WT^ protein showed high binding activity (Fig. 3a). Among the mutants, however, the binding activities of CjWRKY1^Y115F^ and CjWRKY1^Y115L^ were obviously higher than that of CjWRKY1^Y115E^ (Supplementary Fig. S5).

Mutant CjWRKY1^Y115E^-sGFP was transiently expressed in Cj156-S protoplasts to determine the subcellular localization of Tyr-phosphorylated CjWRKY1. CjWRKY1^Y115E^-sGFP protein clearly showed a loss of nuclear localization, in contrast to the wild-type CjWRKY1^WT^-sGFP protein, which was localized in the nucleus (Fig. 3b). On the other hand, the CjWRKY1^Y115F^-sGFP protein showed nuclear localization similar to CjWRKY1^WT^ (Fig. 3b).

**Figure 4:**
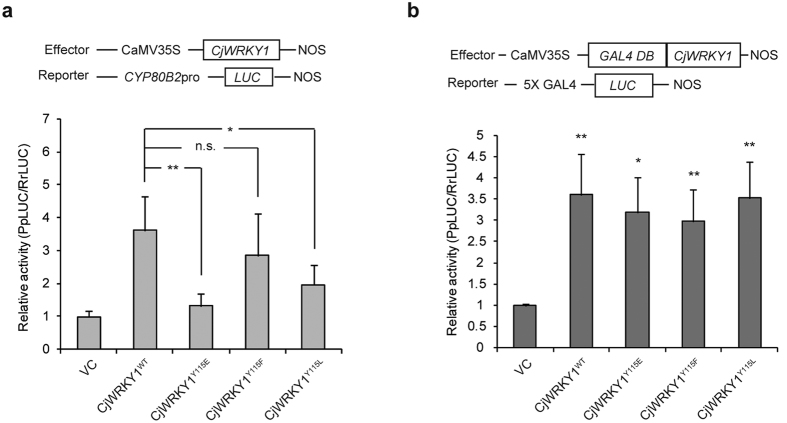
The phosphorylation of CjWRKY1 at Tyr 115 affected the transcriptional activity of the CjWRKY1 protein. (**a**) Transactivation activity of mutant CjWRKY1 proteins detected by dual-LUC reporter assays. The top panel shows the gene organization of the effector and reporter constructs. The bottom panel indicates the relative activity level standardized using the value of the VC as 1. The values are the average of medians of five independent experiments with three biological transfections. The data are represented as the mean ± s.d., *p < 0.05, **p < 0.01, n.s., not significant, Student’s *t*-test. (**b**) Transactivation activity of GAL4DB-fused mutant CjWRKY1 proteins detected as described in (**a**). The values are the average of medians of three independent experiments with three biological transfections. The data are represented as the mean ± s.d., *p < 0.05, **p < 0.01, Student’s *t*-test.

Transcriptional activation of the CjWRKY1 mutants was assayed using reporter constructs containing the promoter region of the *CYP80B2* gene linked to the *Photinus pyralis luciferase* (*LUC*) gene (*CYP80B2*pro::*PpLUC*) in Cj156-S protoplasts. The transactivation activity of CjWRKY1^Y115E^ was much less than CjWRKY1^WT^ (Fig. 4a). On the other hand, CjWRKY1^Y115F^ and CjWRKY1^Y115L^ exhibited considerable transactivation activities compared to CjWRKY1^Y115E^, although their activities were slightly lower than that of CjWRKY1^WT^. Similar results were also observed when the expression of endogeneous BIQ biosynthetic genes was measured in CjWRKY1-expressed Cj156-S protoplasts (Supplementary Fig. S6).

Transcriptional activity using mutants fused with the yeast GAL4 DNA-binding domain (GAL4DB) and a reporter construct with 5 × GAL4 target elements linked to the *LUC* gene to equalize the DNA binding activities of mutant CjWRKY1s, however, indicated that all CjWRKY1 mutants exhibited nearly equal transactivation activities compared to CjWRKY1^WT^ (Fig. 4b). These results suggest that the phosphorylation of CjWRKY1 at Y115 reduced its transcriptional activity by reducing DNA binding to the W-box elements and also via the modification of subcellular localization.

**Figure 5:**
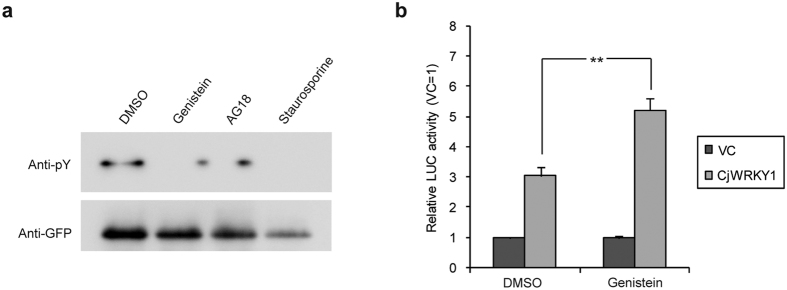
A tyrosine phosphorylation inhibitor enhanced the transactivation activity of CjWRKY1. (**a**) Effect of kinase inhibitors on tyrosine phosphorylation of CjWRKY1. Cj156-S protoplasts were treated with 100 μM genistein, 25 μM AG18, and 0.5 μM staurosporine, and WRKY proteins were co-immunoprecipitated with anti-GFP microbeads. (**b**) Effects of genistein on the transcriptional activation activity of CjWRKY1. A dual-LUC reporter assay was carried out with 0.05% DMSO or 100 μM genistein treatment for 24 h. The relative activity was standardized to the value of each vector control (VC) as 1. The values are the averages of three biological transfections. The above results were confirmed by at least three independent experiments. The data are represented as the mean ± s.d., **p < 0.01, Student’s *t*-test.

Finally, the effect of tyrosine phosphorylation inhibition on the transactivation activity of CjWRKY1 was examined using three tyrosine phosphorylation inhibitors (genistein and AG18 as specific inhibitors for tyrosine phosphorylation and staurosporine as a broad kinase inhibitor), and genistein was the only inhibitor that did not affect protoplast viability (Fig. 5a). As expected, genistein treatment resulted in higher transcriptional activity of CjWRKY1 than DMSO treatment (Fig. 5b). Based on the above results, we also examined the effect of genistein in cultured Cj156-S and CjY cells. However, the expression levels of the genes encoding BIQ biosynthetic enzymes were not changed (Supplementary Fig. S7), suggesting that other types of post-translational regulation of CjWRKY1 are involved in the transcriptional activity of CjWRKY1 in intact cells.

### Degradation of CjWRKY1 and protein phosphorylation

**Figure 6:**
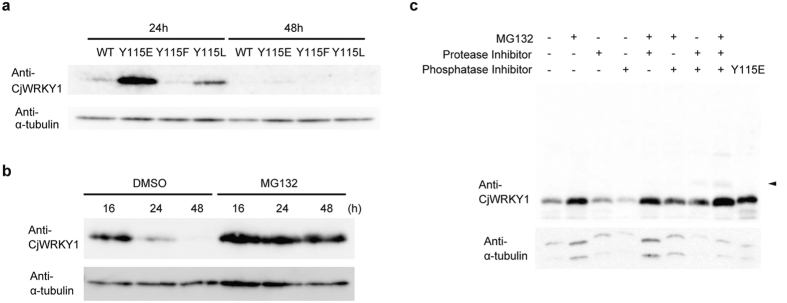
The stability of the CjWRKY1 protein in *C. japonica* 156-S protoplasts. (**a**) The different stabilities of WT and mutant CjWRKY1 proteins. CjWRKY1 proteins were detected using CjWRKY1-specific antibodies. (**b**) A proteasome inhibitor, MG132 (50 μM), stabilized the CjWRKY1 proteins for 48 h. (**c**) Synergistic effects of MG132, protease inhibitors, and protein phosphatase inhibitors on the accumulation of CjWRKY1 proteins. Cj156-S protoplasts were treated with 50 μM MG132, 1 mg/ml complete EDTA-free protease inhibitor cocktail, and/or 5 mg/ml phosSTOP phosphatase inhibitor cocktail. An acrylamide gel containing 50 μM Phos-tag was used to detect phosphorylated WRKY1. Arrows indicate the shifted bands corresponding to phosphorylated CjWRKY1. DMSO (0.1%) was used for mock treatments in (**b**,**c**). All experiments were repeated at least three times with similar results.

Because the Cj156-S and CjY cells showed obvious differences in CjWRKY1 accumulation, we also examined the accumulation of CjWRKY1 mutants in Cj156-S protoplasts. Interestingly, the accumulation levels of mutant CjWRKY1 proteins were higher than those of CjWRKY1^WT^; CjWRKY1^Y115E^ in particular showed the highest accumulation (Fig. 6a, Supplementary Fig. S8). Higher accumulation of mutant CjWRKY1 proteins, however, did not correlate with increased transcriptional activities in the transient reporter assay described above. The different stabilities of CjWRKY1 mutants clearly suggested that post-translational modification was involved in the turnover of CjWRKY1 proteins. A decrease in the accumulation of CjWRKY1 proteins after prolonged incubation also indicated that CjWRKY1 proteins were actively degraded even when post-translational modification was blocked (Fig. 6a).

Treatment with MG132, an inhibitor of the 26S proteasome, indicated that CjWRKY1 was degraded by the ubiquitin-proteasome system (UPS) (Fig. 6b), although the transcript levels of *CjWRKY1* in protoplasts incubated for 48 h were reduced to one-fifth of the levels in protoplasts incubated for 16 h (Supplementary Fig. S9). Because MG132 treatment did not affect the abundance of phosphorylated CjWRKY1, tyrosine phosphorylation may be not involved in the degradation of CjWRKY1 via the UPS or rapid dephosphorylation may occur.

When a phosphatase inhibitor cocktail was added, however, a marked decrease in CjWRKY1 was observed compared to control protoplasts without MG132 and protease inhibitors and also those with MG132. Addition of a protease inhibitor cocktail reduced the degradation of CjWRKY1 enhanced by the phosphatase inhibitors and resulted in a slower migrating band (Fig. 6c). These results suggest that phosphorylated CjWRKY1 protein can be degraded by proteases in the cytosol or in vacuoles.

Whereas MG132 and/or protease inhibitor treatment increased the stability of CjWRKY1 in Cj156-S protoplasts/cells, little enhancement of target biosynthetic gene expression was observed (Supplementary Fig. S10). As discussed below, transcriptional and post-transcriptional regulation of BIQ biosynthesis may be more complicated than expected or these inhibitors may be harmful to diverse processes related to BIQ biosynthesis in *C. japonica* protoplasts/cells.

## Discussion

In this report, we characterized the post-translational modification of CjWRKY1 in the BIQ biosynthesis in *C.japonica*. Our functional characterization of the modification of CjWRKY1 is still not complete, but our data suggest that two types of modification modulate the functional activity of CjWRKY1 in BIQ biosynthesis: Tyr phosphorylation and protein degradation.

In animal cells, protein tyrosine kinases play a crucial role in signal transductions as serine/threonine kinases, whereas the importance of tyrosine phosphorylation has been rather unclear in plant cells because no protein tyrosine kinase homologue was found in *Arabidopsis thaliana* or rice genome^16^,^17^. However, recent phosphoproteome analyses showed comparable tyrosine phosphorylation in plant cells as animal cells, suggesting that tyrosine phosphorylation is also important in plant processes^18^,^19^,^20^. Our results that tyrosine phosphorylation clearly modifies DNA binding activity and subcellular localization of CjWRKY1 in *C. japonica* cells provide the novel evidence on the physiological role of tyrosine phosphorylation in plant cells.

Whereas our results clearly showed the phosphorylation of CjWRKY1 at Y115, the remaining signals detected in immunoprecipitated mutant CjWRKY1^Y115F^-sGFP protein by immunoblot analysis with anti-pY antibodies suggest additional phosphorylation. Recent phosphoproteome analysis indicated that tyrosine phosphorylation preferentially occurred in the conserved domain of phosphoproteins and multiple phosphorylation of tyrosine residues were found in more than 75% of phosphopeptides^18^. Since CjWRKY1 contains nine tyrosine residues except for Y115, additional experiment is needed to study the involvement of other tyrosine residue(s) in the phosphorylation and the regulation of WRKY activity.

On the other hand, several groups have reported that serine/threonine phosphorylation of WRKY proteins by mitogen-activated protein kinases (MAPKs) is important in plant immune responses^14^,^21^,^22^. *Nicotiana benthamiana* WRKY8 was phosphorylated by tobacco MAPKs such as wound-inducible protein kinase (WIPK) and salicylic acid (SA)-inducible protein kinase (SIPK), and the phosphorylation of NbWRKY8 enhanced its DNA binding and trans-activation activities^21^. AtWRKY33, the closest homologue to NbWRKY8 in *A. thaliana*, was also a substrate of AtMPK3/MPK6 and the phosphorylation of AtWRKY33 activated camalexin biosynthesis^22^. Intriguingly, these WRKY proteins belong to Group I WRKY family and have conserved clustered proline-directed serines (SP cluster) at the N-terminal end, the targets of multiple phosphorylation by MAPKs. In addition, *Nicotiana tabacum* WRKY1 and AtWRKY25, which were also phosphorylated by MAPKs *in vitro*, belong to Group I WRKYs^23^,^24^. In contrast, the tyrosine phosphorylation of WRKYGQK core domain was found in CjWRKY1, which belongs to Group IIc. This is first observation of phosphorylation of tyrosine residue comprising WRKYGQK core domain and it is interesting to know whether tyrosine phosphorylation of WRKY domain commonly occurs to inactivate its transcription factor function in Group IIc WRKYs.

On the other hand, UPS-dependent degradation of transcription factors including WRKYs is common in diverse aspects of plant environmental responses and development^25^,^26^,^27^,^28^. For example, *Oryza sativa* WRKY45, which functions in SA signaling in the blast-resistance^29^, was reported to be degraded by the 26S proteasome in the nucleus and to interact with Panicle blast1 (Pb1) protein to enhance plant resistance^27^,^30^,^31^. Considering the alterations in subcellular localization and accumulation levels of mutant CjWRKY1 proteins, especially CjWRKY1^Y115E^, UPS-dependent degradation likely occurs mainly in the nucleus and regulates CjWRKY1 activity in BIQ biosynthesis.

Our results suggest that tyrosine phosphorylation of CjWRKY1 inactivates the expression of genes encoding BIQ biosynthetic enzymes through the reduction of binding activity to promoter sequences and translocation to the cytosol, followed by degradation by proteases in the cytosol or vacuoles (Supplementary Fig. S11). Even without phosphorylation, CjWRKY1 proteins would be rapidly degraded in the nucleus by the 26S proteasome, probably due to the high number of PEST residues^32^. One remaining question is how we can control the turnover of CjWRKY1 to improve BIQ productivity in *C. japonica* cells because the accumulation of CjWRKY1 proteins in CjY cells treated with any inhibitor was also quite low (Supplementary Fig. S10). No additional experiments were successfully able to overcome the low accumulation of CjWRKY1 protein in CjY cells, which show comparable transcript levels in *CjWRKY1* gene and Cj156-S cells^2^. The identification of two CjWRKY1 degradation pathways indicates that BIQ biosynthesis is intensively regulated at a post-translational level by more factors than previously believed. More recently, Nemoto and colleagues reported that *A. thaliana* calcium-dependent protein kinase-related protein kinases (CRKs) can phosphorylate tyrosine residues of β-tubulin proteins as well as several transcription factors including AtWRKY14^33^. Since our expressed sequence tag (EST) prepared from *C. japonica* cells contains several candidate genes encoding protein kinases including a CRK homologue and E3 ligases, it is of interest to identify the interacting partners involved in the phosphorylation and degradation of CjWRKY1 and apply our findings to metabolic engineering of BIQ biosynthesis.

## Materials and Methods

### Plant materials

Suspension-cultured *C. japonica* 156-S cells over-expressing the (*S*)*-scoulerine-9-O-methyltransferase* (*SMT*) gene^34^,^35^ were grown in Linsmaier-Skoog (LS) medium (pH 5.7) containing 3% sucrose, 10 μM 1-naphthylacetic acid (NAA), and 10 nM benzyladenine (BA) on a gyratory shaker (90 rpm) at 23 °C in the dark. Non-selected CjY cells were grown in LS medium (pH 5.7) containing 3% sucrose, 100 μM NAA, and 1 μM BA under the same conditions as Cj156-S cells.

### Vector construction

In addition to a *Cauliflower mosaic virus* (CaMV) 35S::*CjWRKY1* vector constructed in a previous report^5^, the 35S::*CjWRKY1-sGFP* vector was constructed as follows: the *Sal*I/*Nco*I fragment of *CjWRKY1* obtained by PCR was inserted between the 35S promoter and the *NOS* terminator in a pUC18-*sGFP* vector^36^. Mutant-*CjWRKY1* and mutant-*CjWRKY1-sGFP* vectors were generated by inverse PCR-based site-directed mutagenesis using a KOD-plus DNA polymerase (Toyobo), and the constructs were confirmed by nucleotide sequencing.

### Protein extraction from cultured cells

Approximately 1–5 g (FW) of Cj156-S and CjY cells were inoculated in 20 or 100 ml of culture medium and cultured for 1–2 weeks. The grown cells were collected and ground in liquid nitrogen. An equal volume of extraction buffer [50 mM Tris-HCl (pH 7.5), 1% SDS, 10% glycerol, 2 mM DTT, 1 mM EDTA, 50 μM MG132 (Millipore), complete EDTA-free protease inhibitor cocktail (Roche) and phosSTOP phosphatase inhibitor cocktail (Roche)] to the cell weight was added to powdered samples and placed on ice for 10 min. After centrifugation at 15,000 × *g* at 4 °C for 20 min, supernatants were recovered and used as total protein fractions. Protein concentrations were measured using Bio-Rad protein assay dye reagent concentrate (Bio-Rad). Equal amounts of protein samples were separated by 15% SDS-polyacrylamide gel electrophoresis (SDS-PAGE), and the separated proteins were transferred to a polyvinylidene difluoride (PVDF) membrane. After blocking the membrane with 5% skim milk in TTBS buffer [20 mM Tris-HCl (pH 7.6), 137 mM NaCl, and 0.1% Tween-20], the WRKY1 proteins were detected using anti-CjWRKY1 peptide antibodies (prepared by MBL, 1:1,000) and a horseradish peroxidase (HRP)-conjugated anti-rabbit IgG (1:15,000 dilution) in TTBS buffer. The immunoblot signal was detected with ECL Prime Western Blotting Detection Reagent (GE Healthcare) and an ImageQuant LAS4010 (GE Healthcare) or a Chemidoc Touch Imaging System (Bio-Rad). After the first round of immunoblotting, the membrane was stripped and re-used for a second immunoblot assay using an anti-α-tubulin antibody (T5168, Sigma, 1:1,000 dilution) to correct for sample loading.

### PCR analysis

Total RNA was extracted using an RNeasy Plant Mini Kit (Qiagen), and cDNAs were synthesized from 0.2–1 μg of total RNA using a Super Script III (Invitrogen) or a PrimeScript RT reagent kit with gDNA Eraser (TaKaRa). RT-PCR was performed using Go-Taq Master Mix (Promega) under the following conditions: 95 °C for 4 min, followed by the appropriate number of cycles at 95 °C for 15 s, 60 °C for 20 s and 72 °C for 20 s. Real-time PCR was performed using iQ SYBR Green Supermix and a CFX96 Real-Time PCR Detection System (Bio-Rad) under the following conditions: 95 °C for 3 min, followed by 40 cycles of 95 °C for 10 s, 60 °C for 20 s and 72 °C for 20 s. The data were quantified by a standard curve method or the ∆∆Ct method, and the relative expression levels were standardized by the amplification of *ATPase*, *α-tubulin* or *β-actin* as internal controls. The sequences of specific primers are shown in Supplementary Table S2.

### Co-immunoprecipitation (IP) assay

Protoplasts were isolated from Cj156-S cells cultured for 2–3 weeks and transformed using polyethylene glycol (PEG) as previously described with minor modifications^37^. Approximately 80–100 μg of *CjWRKY1-sGFP* plasmids was introduced into approximately 10^6^ protoplasts with 20% (v/v) PEG 4000 solution. After 24 h of culturing on a gyratory shaker (40 rpm) at 24 °C in the dark, the protoplasts were collected at 500× *g* for 3 min and lysed in 500 μl of lysis buffer [50 mM Tris-HCl (pH 7.5), 500 mM NaCl, 10 mM KCl, 10 mM MgCl_2_, 1% Triton X-100, 10% glycerol, 2 mM EDTA, 1 mM DTT, and complete protease inhibitor cocktail, EDTA-free] with two gentle rounds of sonication. After 1 h incubation on a rotator at 4 °C, cell lysate (500 mM NaCl) was diluted to 1/3 with lysis buffer and incubated with 50 μl of anti-GFP microbeads (Miltenyi Biotech) on a rotator for 1 h at 4 °C. The lysate-microbeads mixture was applied to μColumns and eluted after washing according to the manual from the μMACS GFP Tag Protein Isolation Kit (Miltenyi Biotech). Co-immunoprecipitated proteins were analysed with 10% SDS-PAGE and immunoblotted on a PVDF membrane. After blocking the membrane with 5% BSA, phosphorylated proteins were detected using an anti-pY antibody (4G10 platinum, Millipore, 1:1,000 dilution) and a HRP-conjugated anti-mouse IgG (1:10,000–15,000 dilution) antibody in TTBS buffer. The immunoblot signal was detected with ECL Prime Western Blotting Detection Reagent or EzWest Lumi (Atto) and an ImageQuant LAS4010. The same membrane was re-used for the immunoblot assay using anti-GFP antibodies (A6455, Life Technologies, 1:1,000 dilution).

CIP treatment was performed on a μColumn for 40 min at 37 °C before elution. The immunoblot signals were quantified using ImageJ software (http://rsb.info.nih.gov/ij/). Phosphorylation inhibitor treatments were performed in the protoplast culture medium and lysis buffer with 100 μM genistein, 25 μM AG18, or 0.5 μM staurosporine.

### EMSA

GST-CjWRKY1 recombinant proteins were expressed in *Escherichia coli* BL21 (DE3) from full-length cDNAs of *CjWRKY1* or its mutants cloned into pGEX-6P-1 (GE Healthcare) at the *BamH*I and *Not*I sites. Protein expression was induced with 0.1 mM isopropyl-β-D-thiogalactoside (IPTG) for 6 h at 28 °C. GST-CjWRKY1 proteins were extracted from *E. coli* cells and purified using Glutathione Sepharose 4B (GE Healthcare).

The double-stranded DNA probes for EMSA were prepared from biotin-labelled sense (5′-CTGCTGACTAGCTGACTAGCTGACTAGATGG-3′) and antisense (5′-CCATCTAGTCAGCTAGTCAGCTAGTCAGCAG-3′) nucleotides annealed in TEN buffer [10 mM Tris-HCl (pH 8.0), 1 mM EDTA, and 0.1 mM NaCl] for 5 min at 95 °C and cooled at room temperature.

For EMSA, biotin-labelled probes (20 fmol) and the purified recombinant proteins (3 μg) were mixed with 1× binding buffer [1 mM MgCl_2_, 0.5 mM EDTA, 0.05 μg/μl poly (dI-dC)] from a LightShift Chemiluminescent EMSA Kit (Thermo Fisher Scientific). After a 20 min incubation at 4 °C, the reaction mixtures were separated on a 5% polyacrylamide gel and transblotted on Hybond-N+ nylon transfer membranes. The shifts in biotinylated probes were detected using a Chemiluminescent Nucleic Acid Detection Module Kit (Thermo Fisher Scientific) and an ImageQuant LAS4010.

### Subcellular localization

Approximately 10^5^ protoplasts isolated from 2- to 3-week-old Cj156-S cells were transfected with 10 μg of 35S::*CjWRKY1-sGFP* plasmids, and localization of the sGFP fusion proteins was detected using a BZ-9000 microscope (Keyence).

### LUC reporter assay

Transactivation activities of CjWRKY1 mutants were analysed with *CYP80B2* promoter::*PpLUC* (reporter) and 35S::*Renilla reniformis LUC* (reference) vectors as reported previously^5^. In brief, 4 μg of WRKY1 plasmids, 1 μg of reporter plasmids, and 0.025 μg of reference plasmids were co-transfected into protoplasts isolated from 2- to 3-week-cultured Cj156-S cells, and luciferase activities were detected using a Dual-Luciferase Reporter Assay System (Promega) and a Lumat LB9507 luminometer (Berthold) after incubation for 24 h at 24 °C in the dark.

The 35S::*GAL4DB-CjWRKY1* constructs were prepared by insertion of the *CjWRKY1* and mutated *CjWRKY1* fragments into 35S::*GAL4DB* at the *Sma*I and *Sal*I sites. Transactivation activity of the 35S::*GAL4DB-CjWRKY1* constructs was assayed after transfection of 5 μg of effector plasmids, 4 μg of reporter plasmids, and 0.025 μg of reference plasmids into Cj156-S protoplasts. All experiments were performed with three biological transfections.

### Protein extraction from protoplasts

The stabilities of wild-type and mutant CjWRKY1 proteins were evaluated in Cj156-S protoplasts transfected with 10 μg of *35S::CjWRKY1* plasmids. After 20–48 h culture at 24 °C in the dark, the protoplasts were harvested at 500× *g* for 3 min and treated with 50 μl of SDS sample buffer for 5 min at 95 °C. Total protein extracts were separated on a 15% SDS-PAGE gel or 10% SDS-PAGE gel containing 50 μM Phos-tag (Wako). Immunoblot analysis was performed using anti-CjWRKY1 and anti-α-tubulin antibodies as described above. The effects of protease inhibitors and/or phosphatase inhibitors were measured with 50 μM MG132, complete EDTA-free protease inhibitor cocktail, and/or phosSTOP phosphatase inhibitor cocktail.

### Mass spectrometric analysis

Co-IP samples of sGFP and CjWRKY1-sGFP proteins were obtained as described above. Co-immunoprecipitated samples were separated by SDS-PAGE and silver stained (Supplementary Fig. S2). Protein bands were extracted, and proteins were digested in-gel with MS grade modified trypsin (Promega). The extracted peptides were subjected to LTQ LC/MS/MS (Thermo Fisher Scientific). The collected ion spectrum data were analysed with the help of Mr. Y. Watanabe (Proteomic Facility Lab., Grad. Sch. Biostudies, Kyoto Univ.) using MassMatrix version 2.4.2 (www.massmatrix.net/)^38^.

## Additional Information

**How to cite this article**: Yamada, Y. and Sato, F. Tyrosine phosphorylation and protein degradation control the transcriptional activity of WRKY involved in benzylisoquinoline alkaloid biosynthesis. *Sci. Rep.* **6**, 31988; doi: 10.1038/srep31988 (2016).

## Supplementary Material

Supplementary Information
