# Supplementary material for: Tyrosine phosphorylation and protein degradation control the transcriptional activity of WRKY involved in benzylisoquinoline alkaloid biosynthesis
Source: Sci Rep. 2016 Aug 24;6:31988. doi: 10.1038/srep31988 (PMC4995487; doi:10.1038/srep31988)
Supplement: Supplementary Information [file srep31988-s1.pdf]

Supplementary information

Tyrosine phosphorylation and protein degradation control the transcriptional activity of WRKY involved in benzylisoquinoline alkaloid biosynthesis

Yasuyuki Yamada, Fumihiko Sato

Division of Integrated Life Science, Graduate School of Biostudies, Kyoto University, Kyoto, 606-8502, Japan
Corresponding Author: Fumihiko Sato
Email: fsato@lif.kyoto-u.ac.jp

Supplementary Table S1

MassMatrix data

scan#	charge	score	pp	pp2	pptag	m/z	MW(obs)	MW	delta
2953	3	12	6.7	5.7	4	410.31	1228.92	1229.6	-0.69

#	$b^{\wedge+3}$	$b*^{+3}$	b^{+3}	$b^{\wedge++}$	$b*^{++}$	b^{++}	$b^{\wedge+}$	$b*^{+}$	b^{+}	seq	$y^{\wedge+3}$	$y^{\prime+3}$	$y*^{+3}$	y^{+3}	$y^{\wedge++}$	$y^{\prime++}$	$y*^{++}$	y^{++}	$y^{\wedge+}$	$y^{\prime+}$	$y*^{+}$	y^{+}	#
1	55.36		82.02	82.54		122.52	164.07		244.04	Y	383.88	404.54	404.86	410.54	575.32	606.3	606.79	615.31	1149.64	1211.59	1212.58	1229.6	M
2	74.37		101.02	111.05		151.03	221.09		301.06	G		323.53	323.85	329.53		484.79	485.28	493.79		968.56	969.55	986.57	9
3	117.06	138.03	143.71	175.08	206.55	215.06	349.15	412.09	429.12	Q		304.52	304.85	310.52		456.27	456.77	465.28		911.54	912.53	929.55	8
4	159.75	180.73	186.41	239.13	270.6	279.11	477.25	540.19	557.21	K		261.83	262.16	267.84		392.25	392.74	401.25		783.48	784.47	801.49	7
5	183.43	204.41	210.09	274.64	306.11	314.63	548.28	611.22	628.25	A		219.13	219.46	225.14		328.2	328.69	337.2		655.39	656.37	673.4	6
6	216.46	237.44	243.11	324.18	355.65	364.16	647.35	710.29	727.32	V		195.46	195.78	201.46		292.68	293.17	301.68		584.35	585.34	602.36	5
7	259.15	280.13	285.81	388.23	419.7	428.21	775.45	838.39	855.41	K		162.43	162.76	168.44		243.15	243.64	252.15		485.28	486.27	503.29	4
8	297.17	318.15	323.82	445.25	476.72	485.23	889.49	952.43	969.46	N		119.73	120.06	125.74		179.1	179.59	188.1		357.19	358.17	375.2	3
9	335.18	356.16	361.84	502.27	533.74	542.25	1003.53	1066.47	1083.5	N		81.72	82.05	87.72		122.08	122.57	131.08		243.15	244.13	261.16	2
										K		43.71	44.03	49.71		65.05	65.55	74.06		129.1	130.09	147.11	1

Supplementary Table S2

Primer sequences

Primer name	Oligonucleotide sequences (5' to 3')
CjWRKY1-RT-Fw1	TGGAGGAAATATGGGCAAAA
CjWRKY1-RT-Rv1	TGAGCATGCACTCCCTCATA
CjWRKY1-RT-Fw2	GGCAAAAGGCTGTCAAGAAC
CjWRKY1-RT-Rv2	AGAGACGCTGGACTTGCTTC
Cj6OMT-RT-Fw	GTGCATCCTTCACGACTGG
Cj6OMT-RT-Rv	TGCATCATGGATGAGCTTCT
CjCYP80B2-RT-Fw	GAGGTTTTTGAGTTCTGATGTGG
CjCYP80B2-RT-Rv	GGACAATGAGGAGAGGTGGA
Cj4'OMT-RT-Fw	GGAAGGACACCCTGATCAAA
Cj4'OMT-RT-Rv	TTCCTCCACCAACATCAACA
CjCYP719A1-RT-Fw	TGGTGAGGCCACTTCTCTCT
CjCYP719A1-RT-Rv	TCTTGTGCTCCTTGTTCACG
CjbHLH1-RT-Fw	TGCTTCCTCGGTTGCTATCT
CjbHLH1-RT-Rv	TGCATCTATTGGTGCTCCTG
Cjβ-Actin-RT-Fw	GTCACACCGTCCCCATTTA
Cjβ-Actin-RT-Rv	GTCACGGACGATTTCTCGTT
CjATPase-RT-Fw	TCAACAGCCAAAGTTGTTCG
CjATPase-RT-Rv	AATTCAGTCTGCCCGTGATT
Cjα-tubulin-RT-Fw	CAGTGAAACTGGTGCTGGAAAG
Cjα-tubulin-RT-Rv	ATGAGCTGTTCTGGGTGAAACA

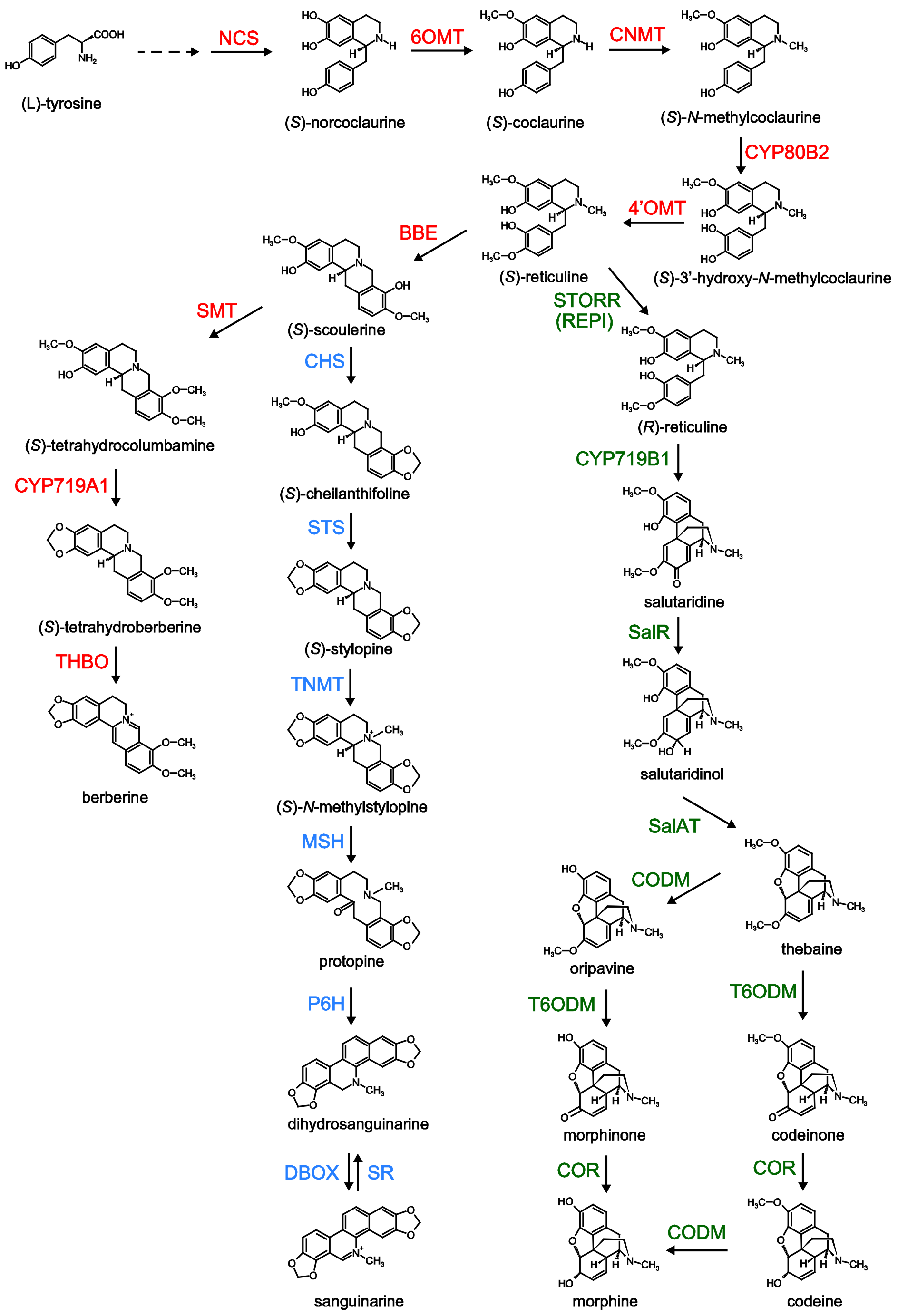

Supplementary Figure S1

BIQ biosynthetic pathway.

Berberine biosynthetic enzymes identified in *C. japonica* are shown in red, and sanguinarine- and morphine-specific biosynthetic enzymes found in *Eschscholzia californica* and *Papaver somniferum* are in blue and green, respectively.

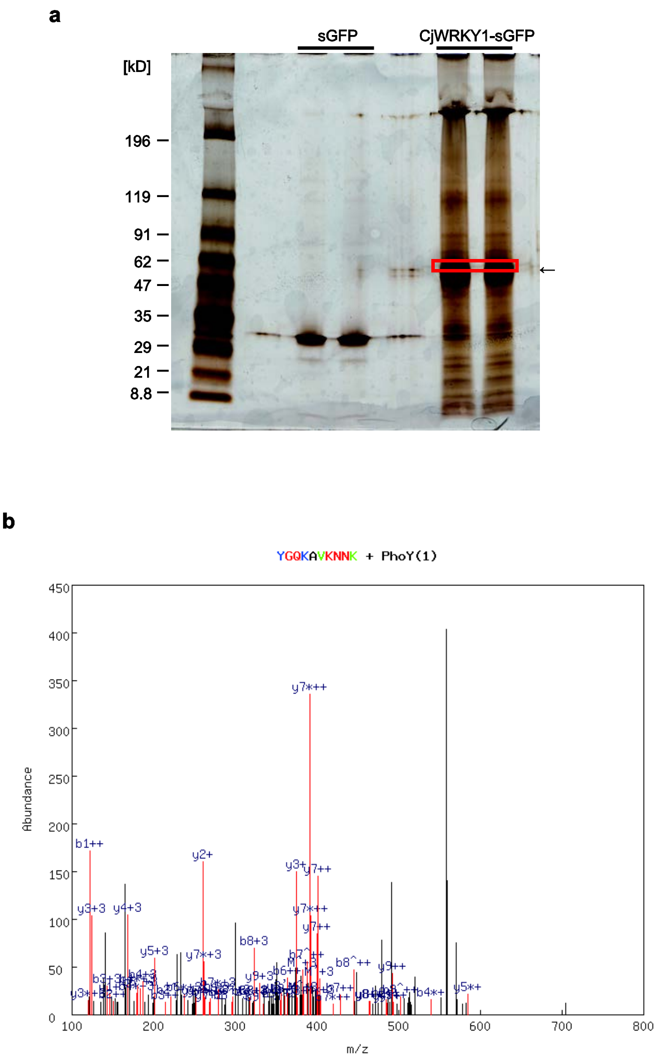

Supplementary Figure S2

Mass fragment analysis of trypsin digests of co-immunoprecipitated CjWRKY1-sGFP proteins.

a, Co-IP samples were separated using 4-15% gradient SDS-PAGE and silver stained. CjWRKY1-sGFP, framed by a red box, was in-gel digested and analysed by MS. b, Product ion spectra of an extracted peptide, YGQKAVKNNK, indicated the tyrosine phosphorylation of the CjWRKY1 protein. The assigned product ions are listed in Supplementary Table S2.

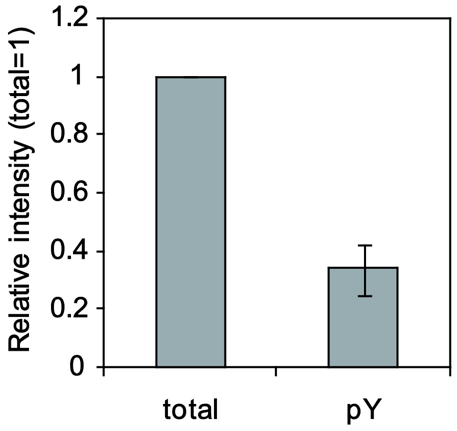

Supplementary Figure S3

Ratio of tyrosine-phosphorylated CjWRKY1-sGFP.

The signal intensity of phosphorylated CjWRKY1-sGFP was quantified by ImageJ software. The value is the average of results from three independent experiments. The data are represented as the mean ± s.d.

a

CYP80B2 **promoter**

TCACGGTTAGGAGGACCAAGACCTAATAAACGATCATTGTTGGACTAAAGCCTAATTACCCTATTATTGGGAGGACCAAAAC

CTAAATAACCTTATTGGTAAGTTTGAATAGGGTCTTTACATATGGGTTTATTGATTTTGATCTATGGATGCATTCCAATGTACA

CATAAGTATTGGTGAAATCGGTAGAGGAAAGAGAGTAGGAAGAAAAGGAAAAGAGTACGTGAGGGAAGGATGAGAGAGAA

AGGAAAAAGGGAGTTATTCTTTTGCCCTTTTTTCTCTAACTTTTCAAGAAAACCGGTGATAAGGGTATCCAGTTTCACAGTAA

TGATGTGGCAATTGCTCATTGGTCTGTTACTTAGTTAGCAGCCAGTAGCTGTCTCCGTACATTTTCGATATACGTATTTGCCG

TACAAATGACTGAATTCACAGTTCCCACGATGGTTGTTGTCCTTTCGCACGGGATGCACGTGAACTTGGAATTAACTTAATT

CCAGCAGCCATCTAGTCAGCAGTTCATTATTCATTAGTCATTTTCGCCCACAGCAAATTTTCTGCCAACAAGCCATAAATAAA

W-box

CAATCATCCGAGTAACCAAAGTGCTTCACATAGTTGCAAGAACGGAACATAGCTGGATAGGAAAAACTATGGAAGTTCTTTC

TATAGCAATTGTTTCCTTTTCGTTCCTCCTCTTCCTCTTCTTCATATTACGCATTCACGCCCTAAGAACCTTCCCCCAGGAC

b

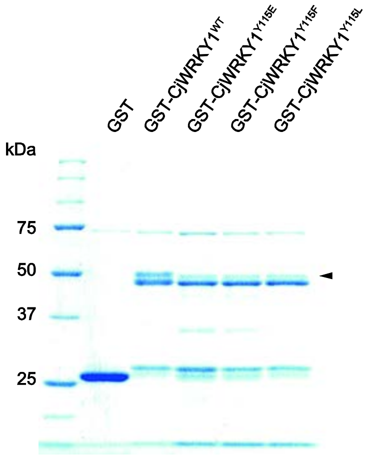

Supplementary Figure S4

The nucleotide sequence of the *CYP80B2* promoter and GST fusion proteins for EMSA.

a, A nucleotide sequence of the W-box element in the *CYP80B2* promoter is shown with a red line. b, The purity of the recombinant GST-CjWRKY1 fusion proteins was analysed by 12% SDS-PAGE and CBB staining. An arrow indicates the GST-CjWRKY1 recombinant proteins.

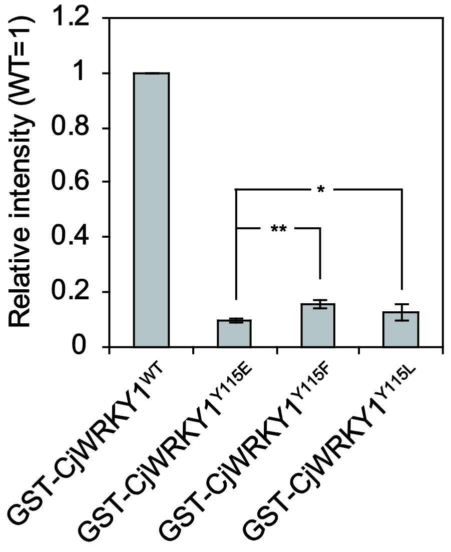

Supplementary Figure S5

Binding activities of mutant CjWRKY1 proteins

The signal intensity of CjWRKY1-DNA complexes in EMSA was quantified by ImageJ software. The value is the average of results from four independent experiments. The data are represented as the mean ± s.d., *p<0.05, **p<0.01, Student's *t*-test.

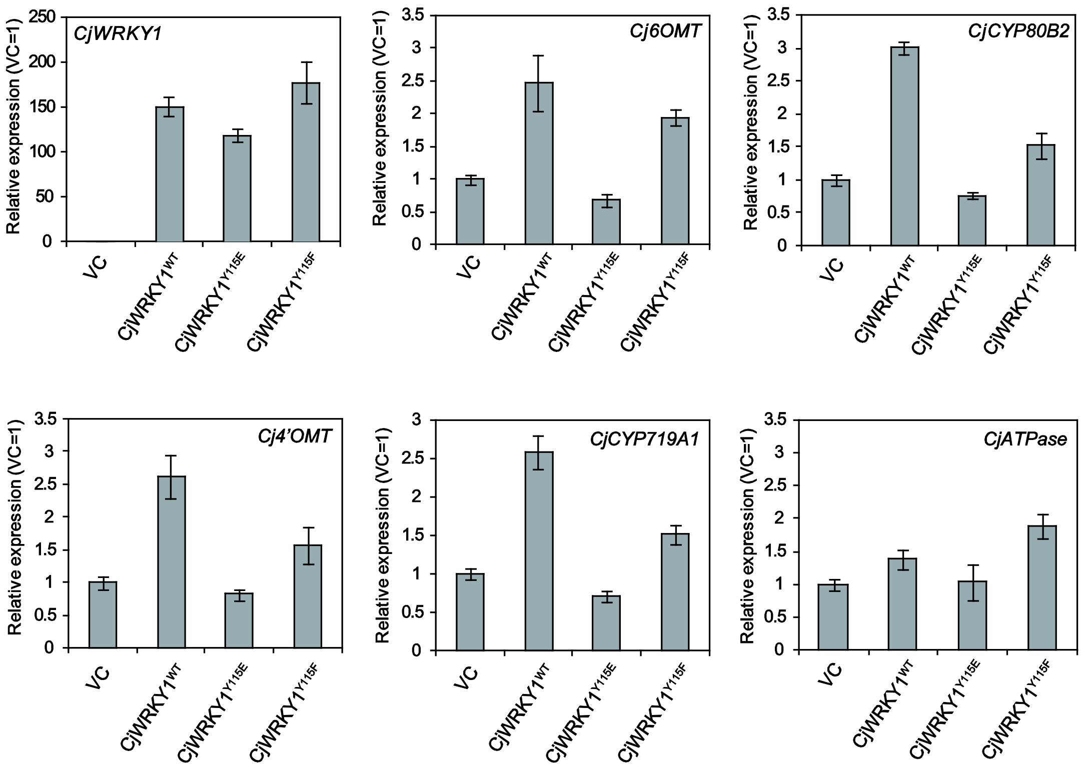

Supplementary Figure S6

Effect of over-expression of mutant CjWRKY1 genes on berberine biosynthetic enzyme genes in Cj156-S cells.

The transcript levels of *CjWRKY1*, *Cj6OMT*, *CjCYP80B2*, *Cj4'OMT*, *CjCYP719A1*, and *CjATPase* were determined by quantitative RT-PCR. The relative expression levels were measured with three technical replicates by the ΔΔCt method and standardized using the *α-tubulin* gene as an internal control. The average value of the DMSO treatment was set as 1. The data are shown as the mean ± s.d.

a

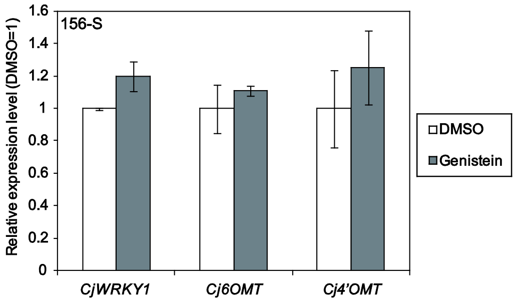

b

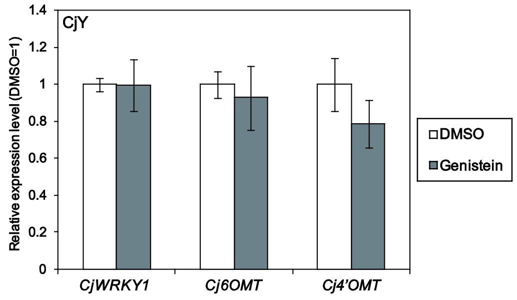

Supplementary Figure S7

Effect of genistein treatment on the expression of berberine biosynthetic enzyme genes in Cj156-S (a) and CjY (b) cells.

The transcript levels of *CjWRKY1*, *Cj6OMT*, and *Cj4'OMT* were determined by quantitative RT-PCR. The relative expression levels were measured with three technical replicates by the ΔΔCt method and standardized using the *β-actin* gene as an internal control. The average value of the DMSO treatment was set as 1. The data are shown as the mean ± s.d. The experiments were repeated three (for a) or two (for b) times to confirm the reproducibility.

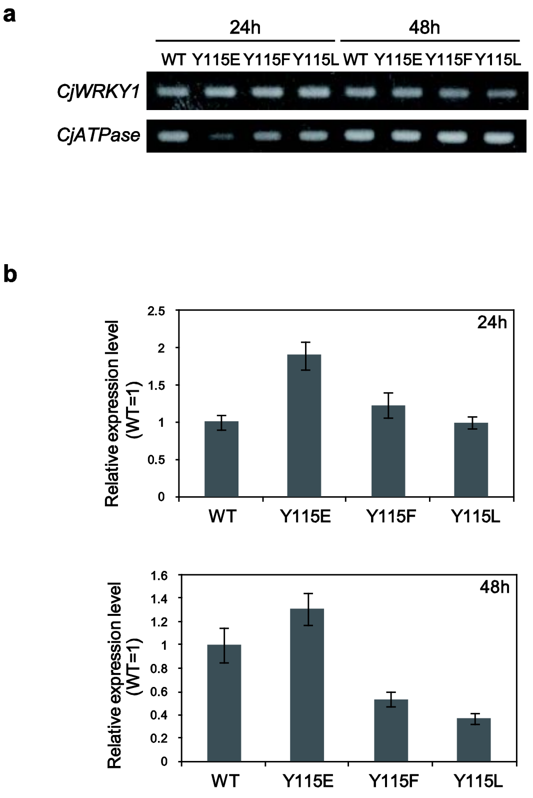

Supplementary Figure S8

Confirmation of equivalent expression levels of the wild-type and mutant *CjWRKY1* genes in Cj156-S protoplasts.

a, Expression of the *CjWRKY1* genes for 48 h was confirmed by RT-PCR. RT-PCR was carried out for 28 (*CjWRKY1*) and 32 (*CjATPase*) cycles. b, Comparison of wild-type and mutant *CjWRKY1* gene expression by real-time PCR. The relative expression levels were quantified by the ΔΔCt method with three technical replicates and standardized using the *α-tubulin* gene as an internal control. The average value of the WT was set as 1. The data are shown as the mean ± s.d.

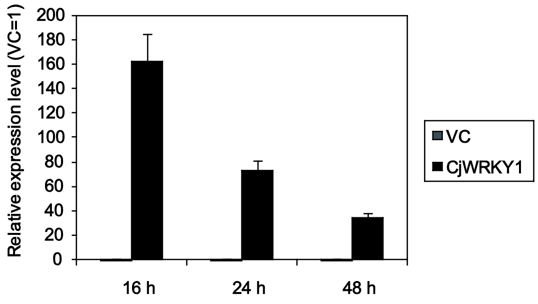

Supplementary Figure S9

Quantification of *CjWRKY1* transcript levels after transient over-expression.

The transcript levels of the *CjWRKY1* gene were measured by quantitative RT-PCR for 16-48 h after transfection of *CjWRKY1* over-expression plasmids. The relative expression levels were quantified by the ΔΔCt method with three technical replicates and standardized with the *ATPase* gene as an internal control. The average value of each VC sample was set as 1. The data are shown as the mean ± s.d.

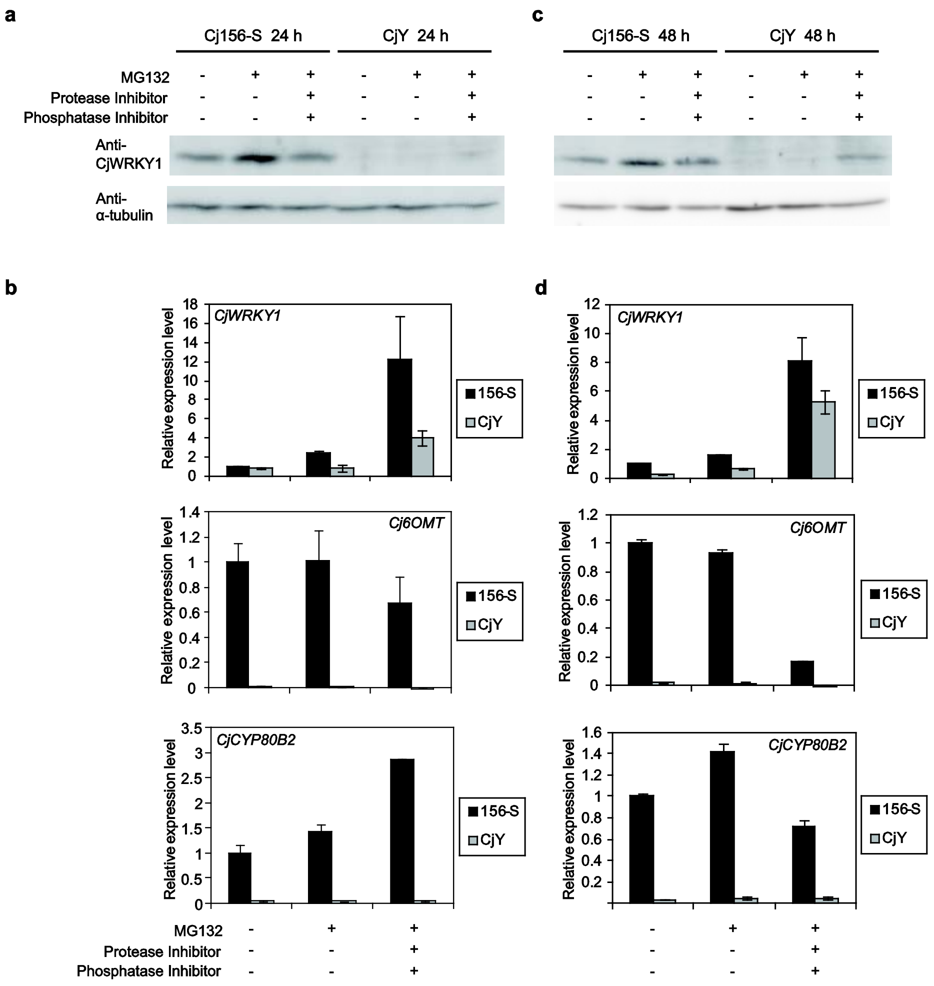

Supplementary Figure S10

The effects of protease and phosphatase inhibitors on the accumulation of CjWRKY1 protein and transcript levels of biosynthetic genes in Cj156-S and CjY cells.

The accumulation of the CjWRKY1 protein was measured at 24 h (a) and 48 h (c) after treatment with 0.1% DMSO, 50 µM MG132, or 50 µM MG132, protease inhibitors and phosphatase inhibitors. The transcript levels of the *CjWRKY1*, *Cj6OMT*, and *CjCYP80B2* genes were measured by quantitative RT-PCR at 24 h (b) and 48 h (d) after treatment. The relative expression levels were estimated by the standard curve method with three technical replicates and standardized with the *β-actin* gene as an internal control. The average value of Cj156-S with DMSO treatment was set as 1. The data are shown as the mean ± s.d.

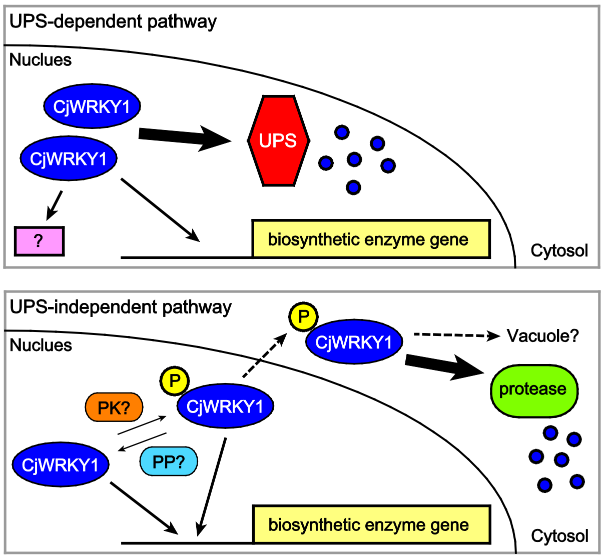

Supplementary Figure S11

Current model of post-translational regulation of the CjWRKY1 protein in *C. japonica* cells.

Top panel; UPS-dependent pathway in which CjWRKY1 is degraded by the 26S proteasome in the nucleus.

Lower panel; UPS-independent pathway in which tyrosine-phosphorylated CjWRKY1 without DNA binding activity is excreted to the cytosol and degraded by unidentified proteases.
